# Association between the generation of cephalosporins for perioperative prophylaxis and postoperative surgical site infections in open fractures: a prospective cohort study

**DOI:** 10.1097/JS9.0000000000002371

**Published:** 2025-04-09

**Authors:** Zhenbang Yang, Hongyu Meng, Junyong Li, Pei Du, Hongzhi Lv, Kuo Zhao, Junzhe Zhang, Ming Li, Zhucheng Jin, Ziheng Peng, Dandan Ye, Kai Ding, Zhaohui Song, Juan Wang, Xin Xing, Yanbin Zhu, Yingze Zhang, Wei Chen

**Affiliations:** a Department of Orthopedic Surgery, The Third Hospital of Hebei Medical University, Shijiazhuang, Hebei, P. R. China; b Department of First Foot and Ankle Surgery, Cangzhou Integrated Traditional Chinese and Western Medicine Hospital, Cangzhou City, Hebei P. R. China; c Department of Orthopedic, Wuxi Hand Surgery Hospital, Wuxi, Jiangsu P. R. China; d Department of Gastroenterology, Xiangya Hospital Central South University, Changsha, Hunan P. R. China

## Abstract

**Background:**

The use of cephalosporins for surgical site infection (SSI) prevention has become a clinical routine, however, high-level evidence regarding the optimal generation for open fractures is currently limited. This study aims to investigate the association between the generation of cephalosporins and SSI risk in open fractures.

**Methods:**

This prospective cohort study used data from the Surgical Site Infection in Orthopedic Surgery (SSIOS), a prospectively maintained database, conducted at a tertiary orthopedic university hospital from October 2014 to December 2020. The primary outcome was occurrence of SSI within 1 year after operation, and its association with the generation of cephalosporins was examined using multivariable logistic regressions and generalized estimating equations. Generalized additive models were used to calculate the relative contribution of potential factors likely influencing SSI.

**Results:**

A total of 3582 eligible patients, 74.6% males, with a mean age of 43.7 ± 14.1 years, were included. First-, second-, and third-generation cephalosporins were used in 1957 (54.6%), 1219 (34.0%), and 406 (11.3%) patients. Compared to first-generation cephalosporins, the use of second-generation cephalosporins was significantly associated with a higher risk of SSI (absolute risk difference [ARD] = 3.70%; 95% CI, 1.90%–5.51%; adjusted OR [aOR] = 1.604; 95% CI, 1.212–2.124), whereas third-generation cephalosporins were not (ARD = 1.02%; 95% CI, −1.78% to 3.82%; aOR = 1.234; 95% CI, 0.790–1.880). Among the 28 potential factors considered, the generation of cephalosporins was ranked 9th in terms of its impact on the risk of SSI.

**Conclusion:**

Perioperative use of higher-generation cephalosporins was not associated with a reduction in postoperative surgical site infections in open fractures. Our study supports existing guidelines that recommend the use of first-generation cephalosporins as the preferred agents for preventing SSIs in open fractures.

**HIGHLIGHTS**

- This prospective cohort study, featuring a large sample size, comprehensive data collection, and thorough adjustment for potential confounders, explores the relationship between the generation of cephalosporin and surgical site infection in Chinese patients with open fracture.
- By quantifying the relative contribution of the generation of cephalosporin among 28 potential factors influencing surgical site infection, this study aids healthcare providers in prioritizing interventions and making informed decisions on antibiotic administration.

## Introduction

Surgical site infection (SSI) is a common and significant complication after open fractures^[^1^]^, often leading to various adverse outcomes (e.g., the need for additional surgeries, amputation, osteomyelitis and increased mortality)^[^2,3^]^, along with substantially increased healthcare costs^[^4^]^. Prophylactic cephalosporins are routinely used worldwide for SSI prevention, as recommended by professional guidelines^[^1,5-7^]^. However, the optimal generation of cephalosporins remains controversial and under-researched. As a result, second- and third-generation cephalosporins were often used arbitrarily by both surgeons and patients in clinical practice, especially in high risk of infection situation, such as open fractures, with a reported application rate as high as 46.4% to 53.0%^[^6,8^]^. Conversely, owing in part to overuse or in some cases misuse of these broad-spectrum cephalosporins, multidrug-resistant (MDR) infections and antibiotic resistance have continued to rise worldwide over the past several decades^[^9^]^.

At present, several major guidelines remain inconsistent in their recommendations regarding the optimal generation of cephalosporins for SSI prevention. The Surgical Infection Society (SIS) guidelines moderately recommend the use of first-generation cephalosporins, such as cefazolin, as prophylactic agents for all Gustilo–Anderson (GA) grades of open fractures (grades I–III)^[^6^]^. In contrast, the Eastern Association for the Surgery of Trauma (EAST) recommended considering broad-spectrum cephalosporins for type III fractures^[^5^]^. However, the EAST guidelines are primarily based on outdated indirect evidence from prospective studies, which did not consider the generation of cephalosporins as a main exposure factor^[^5^]^. Currently, there are no randomized controlled trials (RCTs) directly comparing the effectiveness of different generations of cephalosporins in preventing SSI in open fractures. Two recent observational studies demonstrated non-significant differences in preventing SSI when comparing first- and third-generation cephalosporins for all GA grades of open fractures, but with only a small sample size (n1 = 1149; n2 = 1041) and a short follow-up period (3 months)^[^10,11^]^. Nevertheless, the higher risks of multidrug-resistant bacteria^[^12^]^, renal toxicity^[^13,14^]^, and additional medical costs^[^15,16^]^ associated with higher-generation cephalosporins are repeatedly reported. On the other hand, there exists limited understanding regarding the quantifiable relative contribution of cephalosporin generations of selection amidst a variety of potential factors associated with the risk or mitigation of SSI. Consequently, surgeons are prone to regarding higher-generation cephalosporins as the preferred choice when dealing with challenging cases, potentially contributing to the suboptimal adherence to SIS guideline recommendations, e.g., from 28.5% to 53.6% from literature reports^[^8,17^]^.

This study aims to investigate the association between different generation of cephalosporins and the occurrence of SSI in patients with open fractures, and to quantify its relative importance among a variety of potential risk factors associated with SSI.

## Methods

### Data source

Data were obtained from the *Surgical Site Infection in Orthopedic Surgery (SSIOS)* database, which prospectively collected information on hospitalized patients who underwent orthopedic surgeries since 1 October 2014, and were updated annually. This institution is a university-affiliated tertiary referral teaching hospital in the central province of China, serving a population of over 75.9 million. All patient information was collected manually by a trained team of over 200 individuals. Follow-up was conducted through telephone calls, WeChat (Tencent Holdings Limited, Shenzhen, China), and outpatient visits at 1, 6, and 12 months after discharge. Those who did not respond in two consecutive contacts spaced 24 hours apart were considered lost for follow-up. Double data entry and validation minimized data-entry errors using EpiData software (3.1 for Windows, EpiData Association, Odense, Denmark). The study meets STROCSS 2021 criteria^[^18^]^.

### Study design and populations

**Figure 1.**
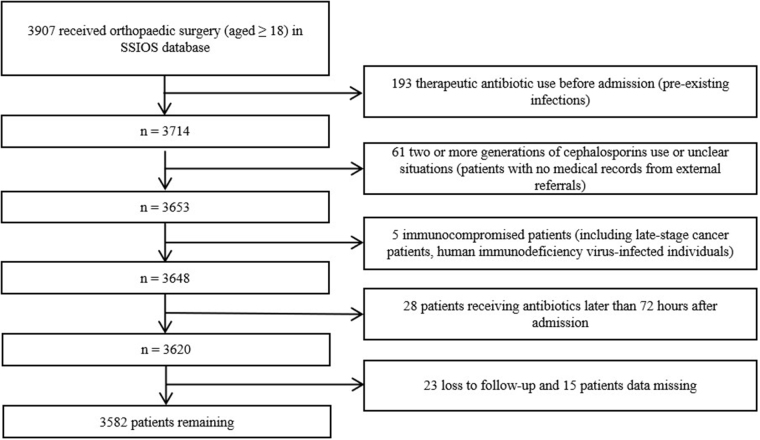
Flow chart showing the patients selection for the cohort.

This prospective cohort study included adult patients from the SSIOS database, spanning October 2014 to December 2020. The exclusion criteria were as follows (Fig. 1): (1) therapeutic antibiotic use before admission (e.g., bacterial pneumonia, urinary tract infection); (2) two or more generations of cephalosporins use or unclear situations (e.g., patients with no medical records from external referrals); (3) immunocompromised patients (including late-stage cancer patients, human immunodeficiency virus-infected individuals); (4) patients receiving antibiotics later than 72 hours after admission; (5) loss to follow-up and missing data. The local ethics committee reviewed and approved the ethical protocol for this study.

### Exposure variable

The main exposure variable was the generation of cephalosporins used perioperatively, determined through double data retrieval from clinical course and physician orders of medical records. Other relevant information simultaneously recorded included the initial timing and duration of cephalosporin prophylaxis, as well as preoperative and postoperative dosage. All cephalosporins were administered via intravenous continuous infusion. Respective proportions of cephalosporins were detailed in the supplementary materials (Table S1, available at: http://links.lww.com/JS9/E68).

### Covariates

Based on prior research and our experience^[^6,19^]^, five main categories of covariates were considered: demographic, medical comorbidity, injury characteristics, surgery data, and laboratory indicators. Demographic covariates included age, sex, place of residence, lifestyle (smoking status, alcohol consumption, and body mass index). Medical comorbidity included as follows: hypertension, diabetes mellitus, cerebrovascular disease, coronary heart disease, chronic kidney disease, chronic liver disease, Charlson Comorbidity Index (CCI), and American Society of Anesthesiologists score. Wound contamination level, GA grade and Injury Severity Score (ISS) score^[^20^]^ were included as injury characteristics. Surgery data included year of surgery, surgeons’ annual surgical volume, surgery duration, emergency surgery, internal fixation, anesthesia method, and type of blood transfusion. Laboratory indicators included red and white blood cell count, albumin, and serum creatinine^[^14^]^. Additionally, we also factored in the initial timing (the time from injury to applying cephalosporins), duration, and dosage of cephalosporins administration as covariates.

### Outcome

Our primary outcome was the incidence of SSI. SSI was defined according to the CDC National Healthcare Safety Network criteria^[^21^]^, diagnosed by infection control physicians based on microbiological tests for inpatients, and confirmed by readmission records or qualified microbiology laboratory cultures results during the one-year follow-up. SSI occurrence timing (pre- or post-discharge) and gram staining of SSI pathogens were recorded simultaneously for subsequent sensitivity analyses. Different cultures isolated from the same episode were included only once if the drug susceptibility test was the same.

### Statistics analyses

Continuous covariables were expressed as mean ± standard deviation (SD) or median [Q1–Q3] depending on normality and compared by one-way analysis of variance (ANOVA) or Kruskal–Wallis H test, as appropriate. Categorical covariables were expressed as numbers (%) and analyzed using the Chi-square test. Missing data that were less than 3% for any variable was addressed using single predictive mean matching (PMM) imputation.

Multiple linear regression was conducted with incidence of SSI as dependent variable to examine any multicollinearity among variables thought to be potential confounders, including covariates above mentioned; and those with a conservative variance inflation factor (VIF) ≥2 were considered indicative of multicollinearity. Both generalized estimating equations (GEE) and multivariable logistic regression models were conducted with generations of cephalosporins as the independent variable and incidence of SSI as the dependent variable. GEE calculated absolute risk differences (% absolute risk difference [ARD], with 95% CIs), adjusting for the covariates after the multicollinearity test^[^22^]^. Multivariable logistic regression models computed the adjusted odds ratios (aOR with 95% CIs), adjusting for covariates with *P* <0.20 in univariable logistic regression analyses, and the goodness-of-fit was evaluated using the Hosmer–Lemeshow test (*P* > 0.05 indicating acceptable result). GAM estimated the relative contribution of the potential risk factors (including generation of cephalosporins) adjusted in GEE to SSI occurrence, as the variance (χ2) minus its degrees of freedom (estimated for spline terms)^[^23^]^, with a higher value indicating a greater contribution.

### Sensitivity analyses

Several sensitivity analyses were performed as follows: (1) Both multivariable logistic regression and GEE were repeated by excluding patients with AST>40 U/L or ALT>56 U/L, ISS scores ≥16^[^24^]^, and undergoing surgery on weekends or holidays^[^25^]^ considering the abnormal liver function, severe trauma and nonrepresentative surgical timing; (2) Considering clustering effect at the surgeon levels (experience and annual surgical volume), GEE and multivariable logistic regression were conducted, with adjustment for them.

Potential population heterogeneity was determined by testing the interaction effects between generations of cephalosporins and the following predefined subgroups: age (<65 vs ≥65), sex, obesity (≥30 vs <30 kg/m^2^), CCI (0 vs 1 vs ≥2), liver function (normal vs AST > 40 U/L or ALT > 56 U/L), renal function (normal vs CREA > 100 μmol/L), GA (grade I vs II vs III), emergency surgery (yes vs no), surgery duration (≤1 vs 1-2 vs 2-3 vs 3-4 vs ≥4 hours) and initial antibiotic timing (<6 vs ≥6 hours). Furthermore, to explore the relationship between generations of cephalosporins and severity and period of SSI, post hoc multinomial logistic regression was used, adjusting for the same variables as in the main MLR models, with the following dependent variables, respectively: (1) MRSA, MDR, and polymicrobial bacterial infection; (2) gram stains of SSI (positive, negative, or both); (3) period of SSI (pre- or post-discharge); (4) inclusion of local antibiotics as an additional adjustment variable; (5) exclusion of patients who received local antibiotic treatment; and (6) the addition of interaction term between cephalosporin generation and GA grade as a new covariate.

All analyses were conducted using R (version 4.2.2). The statistical significance threshold was established at a two-tailed *P* <0.05.

## Results

Of the 3582 patients included, 74.6% were men, and the average age was 43.7 ± 14.1 years; 1957 (54.6%) were administrated with the first-generation cephalosporins, 1219 (34.0%) the second, and 406 (11.3%) the third, respectively. A total of 96 surgeons performed all surgeries, each with a median of 10 surgeries (interquartile range: 2–35). Patients showed statistical baseline differences in CHD, CKD, CCI, ASA, wound class, GA grade, surgery duration, emergency surgery, internal fixation, transfusion type, WBC, ALB, RBC, SAP duration, and preoperative and postoperative antibiotic dosage (*P* < 0.050; Table 1) among the three generations of cephalosporins. 289 cases of patients experienced SSIs, indicating an average infection rate of 8.07%.

**Table 1:** The baseline and characteristics of the cohort

	Overall	First generation	Second generation	Third generation	*P* value
Number	3582	1957	1219	406	-
Gender					
Man	2671	1451(54.3)	916(34.3)	304(11.4)	0.811
Woman	911	506(55.5)	303(33.3)	102(11.2)	
Age	44.0[32.0–54.0]	44.0[31.0–53.0]	43.0[32.0–54.0]	45.5[31.8–54.0]	0.625
<65 years	3297	1782(54.0)	1139(34.5)	376(11.4)	0.050
≥65 years	285	175(61.4)	80(28.1)	30(10.5)	
BMI	24.3[22.0-26.9]	24.5[22.0–26.9]	24.2[22.0–26.4]	24.2[21.5–27.0]	0.076
<30	3342	1819(54.4)	1137(34.0)	386(11.5)	0.296
≥30	240	138(57.5)	82(34.2)	20(8.3)	
Residential place					
Country	2763	1500(54.3)	938(33.9)	325(11.8)	0.326
Urban	819	457(55.8)	281(34.3)	81(9.9)	
Smoking					
Never smoker	2609	1434(55.0)	873(33.5)	302(11.6)	0.772
Current or former smoker	973	523(53.8)	346(35.6)	104(10.7)	
Alcohol consumption					
No	3006	1626(54.1)	1027(34.2)	353(11.7)	0.145
Yes	576	331(57.5)	192(33.3)	53(9.2)	
Hypertension					
No	3149	1708(54.2)	1071(34.0)	370(11.7)	0.095
Yes	433	249(57.5)	148(34.2)	36(8.3)	
Diabetes mellitus					
No	3297	1799(54.6)	1131(34.3)	367(11.1)	0.294
Yes	285	158(55.4)	88(30.9)	39(13.7)	
CVD					
No	3437	1873(54.5)	1180(34.3)	384(11.2)	0.104
Yes	145	84(57.9)	39(26.9)	22(15.2)	
CHD					
No	3189	1715(53.8)	1113(34.9)	361(11.3)	0.006
Yes	393	242(61.6)	106(27.0)	45(11.5)	
CKD					
No	3555	1936(54.5)	1213(34.1)	406(11.4)	0.032
Yes	27	21(77.8)	6(22.2)	0(0.0)	
CLD					
No	3463	1888(54.5)	1178(34)	397(11.5)	0.406
Yes	119	69(58.0)	41(34.5)	9(7.6)	
CCI					
0	2487	1317(53.0)	882(35.5)	288(11.6)	0.037
1	820	485(59.1)	248(30.2)	87(10.6)	
≥2	275	155(56.4)	89(32.4)	31(11.3)	
ISS					
<16	3504	1918 (54.7)	1185 (33.8)	401 (11.4)	0.125
≥16	78	39 (50.0)	34 (43.6)	5 (6.4)	
WBC	8.45[6.54–10.85]	8.29[6.50–10.70]	8.66[6.67–11.21]	8.40[6.39–10.88]	0.042
ALB	39.8[35.0–43.9]	40.3[35.5–44.3]	39.1[34.2–43.1]	39.6[35.1–43.4]	<0.001
RBC	4.14[3.55–4.66]	4.19[3.58–4.70]	4.04[3.47–4.59]	4.13[3.59–4.67]	<0.001
ALT/AST					
Normal value	2914	1604(55.0)	977(33.5)	333(11.4)	0.414
Abnormal value	668	353(52.8)	242(36.2)	73(10.9)	
CREA					
Normal value	3545	1932(54.5)	1212(34.2)	401(11.3)	0.149
Abnormal value	37	25(67.6)	7(18.9)	5(13.5)	
Emergency surgery					
No	1907	1124(58.9)	599(31.4)	184(9.6)	<0.001
Yes	1675	833(49.7)	620(37)	222(13.3)	
Internal fixation					
Yes	1455	837(57.5)	444(30.5)	174(12.0)	<0.001
No	2127	1120(52.7)	775(36.4)	232(10.9)	
Anesthesia type					
General	2870	1562(54.4)	969(33.8)	339(11.8)	0.190
Others	712	395(55.5)	250(35.1)	67(9.4)	
ASA					
I	1065	577(54.2)	326(30.6)	162(15.2)	<0.001
II	2135	1189(55.7)	738(34.6)	208(9.7)	
III	357	182(51)	142(39.8)	33(9.2)	
IV & V	25	9(36.0)	13(52.0)	3(12.0)	
Wound class					
Clean	1752	946(54)	619(35.3)	187(10.7)	0.003
Clean-contaminated	1266	664(52.4)	440(34.8)	162(12.8)	
Contaminated & dirty	564	347(61.5)	160(28.4)	57(10.1)	
Surgery duration					
≤60 min	382	222(58.1)	111(29.1)	49(12.8)	0.004
61–119 min	1429	806(56.4)	449(31.4)	174(12.2)	
120–179 min	889	477(53.7)	329(37)	83(9.3)	
≥180 min	882	452(51.2)	330(37.4)	100(11.3)	
Transfusion type					
None or autologous	3179	1759(55.3)	1050(33.0)	370(11.6)	0.001
Allogeneic or/and autologous	403	198(49.1)	169(41.9)	36(8.9)	
Years					
2014	75	45(60.0)	24(32.0)	6 (8.0)	0.824
2015	474	257(54.2)	162(34.2)	55(11.6)	
2016	594	322(54.2)	189(31.8)	83(14.0)	
2017	746	408(54.7)	260(34.9)	78(10.5)	
2018	703	381(54.2)	246(35.0)	76(10.8)	
2019	641	356(55.5)	214(33.4)	71(11.1)	
2020	349	188(53.9)	124(35.5)	37(10.6)	
GA grade					
I	272	174(64.0)	71(26.1)	27(9.9)	<0.001
II	1865	1076(57.7)	574(30.8)	215(11.5)	
III	1445	707(48.9)	574(39.7)	164(11.3)	
Antibiotic timing (h)	3[3–6]	3[3–6]	3[3–6]	4[3–6]	0.282
SAP duration (d)	4[2–5]	3[2–5]	4[2–6]	4[2–5]	0.004
Preoperative antibiotic dosage (g)	2[2–2]	2[2–2]	2[2–2]	2[2–2]	<0.001
Postoperative antibiotic dosage (g)	4[4–4]	4[4–4]	4[4–4]	4[4–4]	<0.001

BMI = body mass index; CVD = cerebrovascular disease; CHD = coronary heart disease; CKD = chronic kidney disease; CLD = chronic liver disease; WBC = white blood cell; RBC = red blood cell; ASA = American Society of Anesthesiologist score; GA = Gustilo–Anderson; SAP duration = duration of surgical antibiotic prophylaxis.

All continuous variables were described by mean ± SD; enumeration data were presented by percentage (%).

Multicollinearity among all covariables was not observed (Supplementary Table S2, available at: http://links.lww.com/JS9/E68). As shown in Table 2, second-generation cephalosporins were significantly associated with a higher risk of SSI compared to first-generation cephalosporins (ARD = 3.70%; 95% CI, 1.90%–5.51%; unadjusted OR = 1.841; 95% CI, 1.426–2.380; adjusted OR [aOR] = 1.604; 95% CI, 1.212–2.124), whereas third-generation cephalosporins were not (ARD = 1.02%; 95% CI, −1.78% to 3.82%; unadjusted OR = 1.276; 95% CI, 0.839–1.888; aOR = 1.234; 95% CI, 0.790–1.880). In the relative contribution of factors leading to SSI (Table 3), the generation of cephalosporins was ranked 9th and was statistically significant (*P* < 0.050).

**Table 2:** Logistics regression between the generation of cephalosporins and surgical site infection

Cephalosporin generation	No.(%)	Absolute risk difference (95% CI)% a	*P* value	Unadjusted logistic regression analyses, OR (95% CI)	*P* value	Adjusted logistic regression analyses, OR (95% CI)^b^	*P* value
First	123/1957 (6.3%)	1.00 [Ref]		1.00 [Ref]	-	1.00 [Ref]	-
Second	134/1219 (11.0%)	3.70 (1.90–5.51)	0.002	1.841 (1.426–2.380)	<0.001	1.604 (1.212–2.124)	0.001
Third	32/406 (7.9%)	1.02 (−1.78 to 3.82)	0.507	1.276 (0.839–1.888)	0.238	1.234 (0.790–1.880)	0.341

^a^ Expressed as a percentage (with 95% CI) calculated using generalized estimating equations by adjusting the variables: cephalosporin generation, gender, age, BMI, antibiotic timing, residential place, smoking, alcohol use, hypertension, diabetes mellitus, cerebrovascular disease, coronary heart disease, chronic kidney disease, chronic liver disease, WBC, ALB, RBC, emergency surgery, internal type, anesthesia type, American Society of Anesthesiologist score, wound class, surgery duration, transfusion type, preoperative antibiotic dosage, postoperative antibiotic dosage, SAP duration, and GA grade.

^b^ Included variables with *P* <0.20 from univariate analysis into binary logistic regression analysis: cephalosporin generation, age, BMI, residential place, diabetes mellitus, coronary heart disease, emergency surgery, antibiotic timing, internal type, anesthesia type, American Society of Anesthesiologist score, Surgery Duration, WBC, ALB, RBC, transfusion type, GA grade, and SAP duration.

[Ref] indicated the reference group.

**Table 3:** Relative contribution value of the infection rate

Variables	χ^2^-*df*	Rank	Variables	χ^2^-*df*	Rank	Variables	χ^2^-*df*	Rank
Gender	−0.8	20	CKD	−1	26	GA grade	9.0	8
Age	1.1	14	CLD	−0.8	21	Surgery duration	12.3*	4
BMI	−0.7	19	WBC	5.6*	10	Transfusion type	9.2*	6
Residential place	9.3*	5	ALB	25.5*	2	Cephalosporin generation	5.8*	9
Smoking	−0.8	22	RBC	−0.9	23	SAP duration	−0.9	24
Alcohol use	−0.9	25	Emergency surgery	3.3*	13	Antibiotic timing	28.4	1
Hypertension	−0.2	17	Internal type	−0.3	18	Preoperative antibiotic dosage	4.5*	12
Diabetes mellitus	4.9*	11	Anesthesia type	−1.0	28	Postoperative antibiotic dosage	0.2	16
CVD	−1.0	27	ASA	1.0	15			
CHD	16.2*	3	Wound class	9.0*	7			

Continuous variables were fitted by penalized thin plate regression splines, while categorical variables were treated as factors; the maximum likelihood (ML) method was used, with the model framework specified as Poisson regression.

BMI = body mass index; CVD = cerebrovascular disease; CHD = coronary heart disease; CKD = chronic kidney disease; CLD = chronic liver disease; WBC = white blood cell; RBC = red blood cell; ASA = American Society of Anesthesiologist score; SAP duration = duration of surgical antibiotic prophylaxis.

^*^ *P* < 0.05.

**Figure 2.**
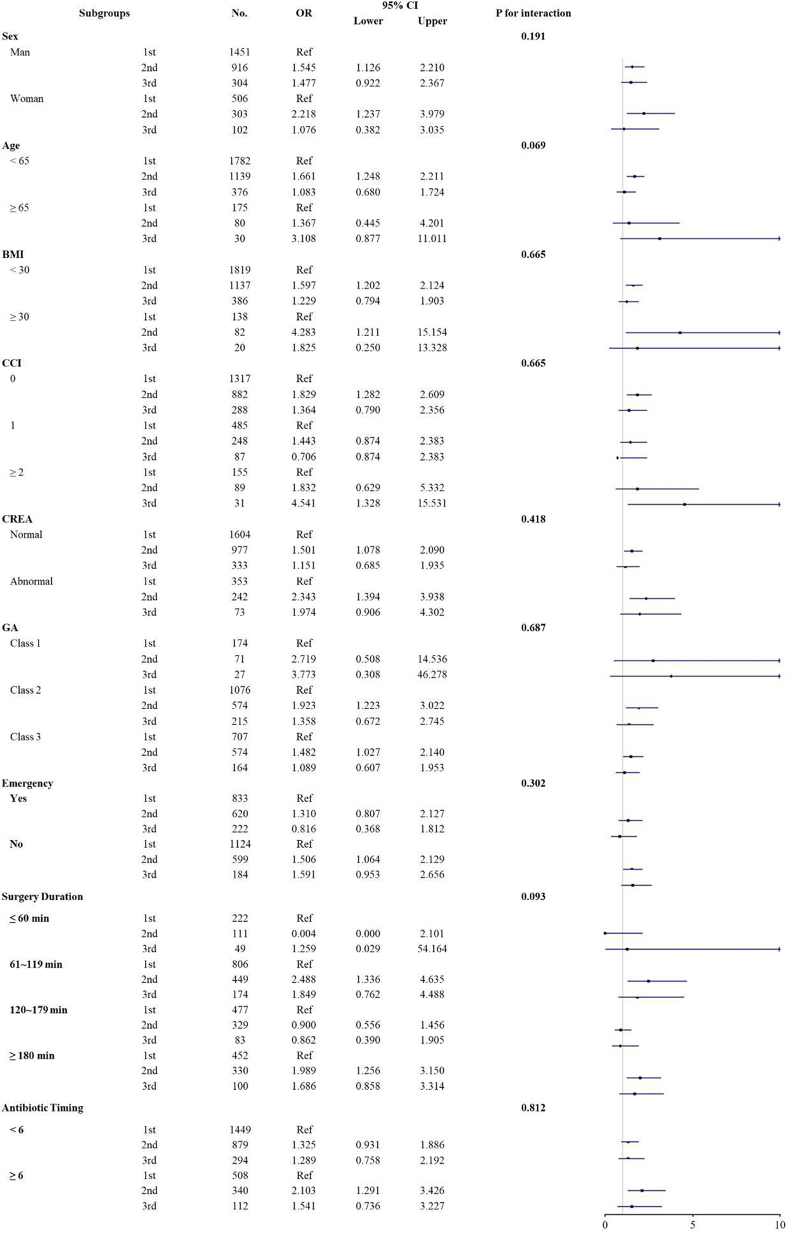
The association of generation with the risk of SSI in various subgroups.

Our main results remained robust in the sensitivity analyses (Supplementary Tables S3–S4 and S6, available at: http://links.lww.com/JS9/E68). No significant interaction effects were observed between generations and age, CCI, GA grade, antibiotic timing or other terms listed (*P* for interaction > 0.050; Fig 2). Second-generation cephalosporins were significantly associated with a higher risk of Gram-positive bacterium infection (aOR = 1.915; 95% CI, 1.240–2.957) and predischarge infection (aOR = 1.690; 95% CI, 1.271–2.246; Table 4). Third-generation cephalosporins significantly increased the risk of polymicrobial infection (aOR = 2.831; 95% CI, 1.168–6.860; Table 4), and post-discharge SSI (aOR = 3.408; 95% CI, 1.384–8.392; Table 4). In post-hoc analyses, compared to first-generation cephalosporins, second-generation cephalosporins showed an increased risk of MDR infection (ARD = 2.06%; 95% CI, 1.27%–2.84%; aOR = 2.004; 95% CI, 1.194–3.399; Supplementary Tables S5 and S7, available at: http://links.lww.com/JS9/E68).

**Table 4:** Associations of cephalosporin generation with gram staining characteristics of infectious agents (multinomial logistic regression)

Model	Unadjusted logistic regression analyses, OR (95% CI)	*P* value	Adjusted logistic regression analyses, OR (95% CI)	*P* value
Model 1^a^				
Gram negative				
First	1.00 [Ref]	-	1.00 [Ref]	-
Second	1.954 (1.387–2.754)	<0.001	1.672 (1.164-2.401)	0.005
Third	1.303 (0.754–2.249)	0.343	1.273 (0.723-2.241)	0.403
Gram positive				
First	1.00 [Ref]	-	1.00 [Ref]	-
Second	1.848 (1.213–2.813)	0.004	1.915 (1.240-2.957)	0.003
Third	0.798 (0.356–1.788)	0.584	0.863 (0.380–1.963)	0.726
Polymicrobial b				
First	1.00 [Ref]	-	1.00 [Ref]	-
Second	1.373 (0.658–2.866)	0.398	1.426 (0.666–3.050)	0.361
Third	2.452 (1.042–5.771)	0.040	2.831 (1.168–6.860)	0.021
Model 2^a^				
Predischarge infection				
First	1.00 [Ref]	-	1.00 [Ref]	-
Second	1.894 (1.446–2.481)	<0.001	1.690 (1.271–2.246)	<0.001
Third	1.090 (0.691–1.719)	0.712	1.092 (0.680–1.755)	0.715
Post-discharge infection				
First	1.00 [Ref]	-	1.00 [Ref]	-
Second	1.465 (0.694–3.090)	0.316	1.589 (0.733–3.446)	0.241
Third	2.615 (1.101–6.213)	0.029	3.408 (1.384–8.392)	0.008

Adjust variables: Model 1: cephalosporin generation, DM, CHD, emergency surgery, internal fixation, ASA, transfusion type, WBC, ALB, RBC, antibiotic timing, and surgery duration. Model 2: cephalosporin generation, DM, CHD, emergency surgery, ASA, transfusion type, WBC, ALB, antibiotic timing, and surgery duration.

Abbreviation: DM = diabetes mellitus; CHD = coronary heart disease; ASA = American Society of Anesthesiologist score; WBC = white blood cell; ALB = albumin; RBC = red blood cell.

^a^ The reference category is no infection.

^b^ Both gram-negative and gram-positive bacterial infection.

[Ref] indicated the reference group.

## Discussion

This prospective population-based cohort study, conducted among open fracture patients receiving cephalosporins as the drug of choice for SAP perioperatively, found that higher-generation (second and third) cephalosporins did not further reduce the risk of SSI within one year after surgery compared with first-generation cephalosporins; instead, second-generation cephalosporins were associated with an increased risk of SSI. Additionally, our study found that second- and third-generation cephalosporins were associated with an increased risk of gram-positive bacteria and predischarge infections, as well as polymicrobial and post-discharge infections, respectively.

The finding that higher-generation cephalosporins do not confer additional reductions in SSIs following open fracture surgeries may be beyond the expectations of both clinicians and patients. This can be explained by several factors as follows. First, compared to first-generation cephalosporins, higher-generation cephalosporins theoretically have a narrow antimicrobial spectrum against gram-positive bacteria, which account for 37.8% to 62.5% (46.4% in our study) of total infections in open fractures^[^26,27^]^, limiting their effectiveness in preventing SSI. Second, in clinical practice, around 27.3% to 82.6%^[^28,29^]^ (55.7% in our study) of gram-negative infections in open fractures are caused by bacteria that exhibit low susceptibility or even antibiotic resistance to high-generation cephalosporins (e.g., *Pseudomonas aeruginosa, Acinetobacter baumannii, Enterobacter cloacae*), which differs from antibiotic susceptibility of the same strains with non-resistance in the laboratory^[^30,31^]^, indicating the unreliability of laboratory-verified antimicrobial spectra in actual clinical applications^[^32^]^. Third, intravenous antibiotics are delivered to tissues with sufficient blood supply, but this pathway is more likely to be compromised in acutely injured tissues or blood vessel during open fractures. In this case, even if the serum concentration of the drug is well above the minimum inhibitory concentration (MIC), cephalosporins of any generation may fail to achieve the MIC in localized tissues with poor blood supply, thus failing to exert their theoretical antibacterial effect^[^33,34^]^. Fourth, hypoalbuminemia (ALB < 3.5 g/dL) is relatively common in open fractures (incidence ranging from 40% to 50%, our study is 25%), which may increase the clearance of some higher-generation cephalosporins (e.g., ceftriaxone), leading their relatively insufficient of effective concentration^[^35^]^. Additionally, higher-generation cephalosporins may not penetrate the damaged skin, exposed bone fragments, blood clots, foreign materials such as soil, and bacterial biofilms, thus failing to eradicate the bacteria within these foreign materials^[^36^]^.

Currently, clinical guidelines or general consensus agree that first-generation cephalosporins are the optimal choice for GA grade I and II open fractures, but there is still controversy regarding GA grade III fractures. AAOS^[^1^]^ and EAST^[^5^]^ guidelines both recommended that gram-negative coverage should be added for GA grade III open fractures; however, this recommendation is primarily based on indirect evidence from an outdated prospective studies^[^37^]^. In contrast, the latest SIS guidelines^[^6^]^, published in 2022, recommend against the use of extended-spectrum antibiotics due to the insufficient evidence. Our subgroup analysis, stratified by GA classification (Fig. 2), shows that for GA grade III open fractures, higher-generation cephalosporins do not further reduce the incidence of SSI (*P* for interaction > 0.050), supporting the recommendations of the SIS guidelines. However, this result should be interpreted with caution due to multiple comparisons and the relatively small number of GA grade III open fractures included. Future studies specifically focusing on GA grade III open fractures are needed to address this critical issue.

Considering the potential outlier-driven bias in effect estimation, several sensitivity analyses were performed by excluding the abnormal liver function, severe trauma, nonrepresentative surgical timing, and adjusting for the clustering effect at surgeon levels, but without significant or substantial change in effect size. Previous studies comparing the pharmacodynamics and pharmacokinetics of different generations have shown that cephalosporins efficacy is significant influenced by age, kidney function^[^38,39^]^, and injury severity^[^5^]^. However, our subgroup analyses based on these factors failed to identify any significant interaction (Fig. 2). These consistent results supported the robustness of our primary findings. Additionally, compared to first-generation cephalosporins, our exploratory analyses showed that second-generation were associated with an increased risk of MDR infections (Table S5), which might be explained by their broader selective pressure leading to resistant bacteria becoming the dominant population. Second-generation cephalosporins showed a higher risk in both SSI and MDR infections, rather than a protective effect. Further research is therefore warranted to elucidate biological plausibility responsible for the finding and which specific subgroups may benefit from this mechanic. Still, care should be taken in interpreting these findings because they are from post-hoc analyses, include multiple comparisons, and, in some cases, exhibit wide confidence intervals.

Our study shows that among all included variables, the relative contribution of generation ranks 9th (middle third), suggesting that generation of cephalosporins was a relatively unimportant factor for the occurrence of SSI. This finding provides updated evidence for the current guidelines that recommend the use of first-generation cephalosporins as adequate for preventing SSI in open fractures^[^6^]^. However, improving adherence to the guidelines remains a significant challenge in practice, as the current guidelines compliance ranges only from 28.5% to 53.6%^[^8,17^]^, despite the issuance of the most recent guidelines over 6 years. The present result may help improve guideline adherence by prompting healthcare providers and researchers to re-evaluate the role of cephalosporin generations in preventing SSI. Additionally, to improve compliance and reduce unnecessary use of broad-spectrum antibiotics, we have proposed several actionable strategies. First, for non-recommended antibiotic prescriptions, clinicians should provide explicit written justification in the medical records. Second, healthcare service leaders should audit and provide feedback on clinicians’ antibiotic prescriptions, comparing their broad-spectrum antibiotics usage percentages with that of their peers. Finally, we recommend provided face-to-face educational training to patients when admitted, using effective communication strategies to educate them on when broad-spectrum antibiotics are and when are not needed, as well as to raise awareness about the potential harms of broad-spectrum antibiotic treatment.

## Clinical implication

There is ongoing debate in current clinical guidelines regarding the use of broad-spectrum antibiotics (including coverage for Gram-negative bacilli or *Clostridium difficile*) in the management of severe open fractures (GA grade III). Our findings suggest that the use of higher-generation cephalosporins not only fall to further reduce SSI but may, in fact, increase the risk of MDR infections. Additionally, the importance of cephalosporin generation was not as significant as clinicians had expected, with its ranking at 9th among 28 potential risk factors, which might inform better clinical guidelines. Based on the data presented, theoretically, a 45.3% reduction in the use of broad-spectrum cephalosporins is possible, which aligns with the estimates reported by Lin *et al*^[^8^]^. However, in practice, this may present a significant challenge. In 2019, an international survey of orthopedic surgeons registered with AO Trauma found that up to 40% of 1,197 respondents believed that broad-spectrum antibiotic regimens should be used to prevent SSI in open fractures^[^40^]^. Future high-quality studies are needed to confirm our findings and elucidate the pathophysiological or biological mechanisms underlying the increased risk of MDR associated with higher-generation cephalosporins.

### Limitation

Our study has several limitations. First, we used a conservative definition for SSI confirmed by positive bacterial cultures, which likely underestimated the incidence since some infections may not be cultured or may result in false negatives. Instead, relying on clinical symptoms and signs of infection as diagnostic criteria may overestimate SSI rate as these symptoms are nonspecific and may also occur in some non-infectious conditions (e.g., edema, fat liquefaction, postoperative hematoma)^[^41^]^, especially for less experienced doctors. Besides, the SSI rate in this study is consistent with the previous studies that used same definition^[^42,43^]^. Second, the proportion of cephalosporins was not well distributed in each generation, which may affect the accuracy of interpreting the exposure-outcome relationship due to intrinsic differences within a generation (e.g., pharmacokinetics, pharmacodynamics). Third, our subgroup analyses showed that third-generation cephalosporins had no statistically significant protective effect against SSI compared to first generation for GA III fractures. However, this result should be interpreted with caution due to the underpowered small sample size (n = 164). Additional prospective studies are needed to determine the effect of the use of prophylactic Gram-negative antibiotic coverage in GA III fractures. Fourth, several unknown or unmeasured factors (e.g., degree of soft tissue injury, complexity of fracture pattern, the interval from injury to irrigation and debridement, surgical disinfection methods) may represent potential sources of residual confounding. Finally, the single-center design of this study conducted in a tertiary referral hospital in China may limit its generalizability to other populations and healthcare settings, particularly in areas with distinct antibiotic resistance patterns or clinical practices, despite our efforts in controlling for confounders and ensuring the robustness of the results through extensive sensitivity and subgroup analyses.

## Conclusion

Perioperative use of higher-generation cephalosporins was not associated with a reduction in postoperative SSIs in open fractures. This study supports existing guidelines that recommend the use of first-generation cephalosporins as the preferred agents for preventing SSIs in open fractures.

## Data Availability

Due to our hospital policy, our data cannot be freely disclosed. For inquiries, please contact Yanbin Zhu.

## Research registration unique identifying number (UIN)

Registry: Chinese Clinical Trial Registry (ChiCTR) Registration number: ChiCTR-PPC-14005360

https://www.chictr.org.cn/searchproj.html?title=&officialname=&subjectid=&regstatus=&regno=ChiCTR-PPC-14005360&secondaryid=&applier=&studyleader=&createyear=&sponsor=&secsponsor=&sourceofspends=&sudyailment=&studyailmentcode=&studytype=&studystage=&studydesign=&recruitmentstatus=&gender=&agreetosign=&measure=&country=&province=&city=&institution=&institutionlevel=&intercode=&ethicalcommitteesanction=&whetherpublic=&minstudyexecutetime=&maxstudyexecutetime=&btngo=btn.
